# Supplementary material for: Combination antagonism of TNF superfamily signaling for T cell immunosuppression
Source: Front Immunol. 2026 Jul 23;17:1867995. doi: 10.3389/fimmu.2026.1867995 (PMC13442446; doi:10.3389/fimmu.2026.1867995)
Supplement: Supplementary file 1 [file DataSheet1.pdf]

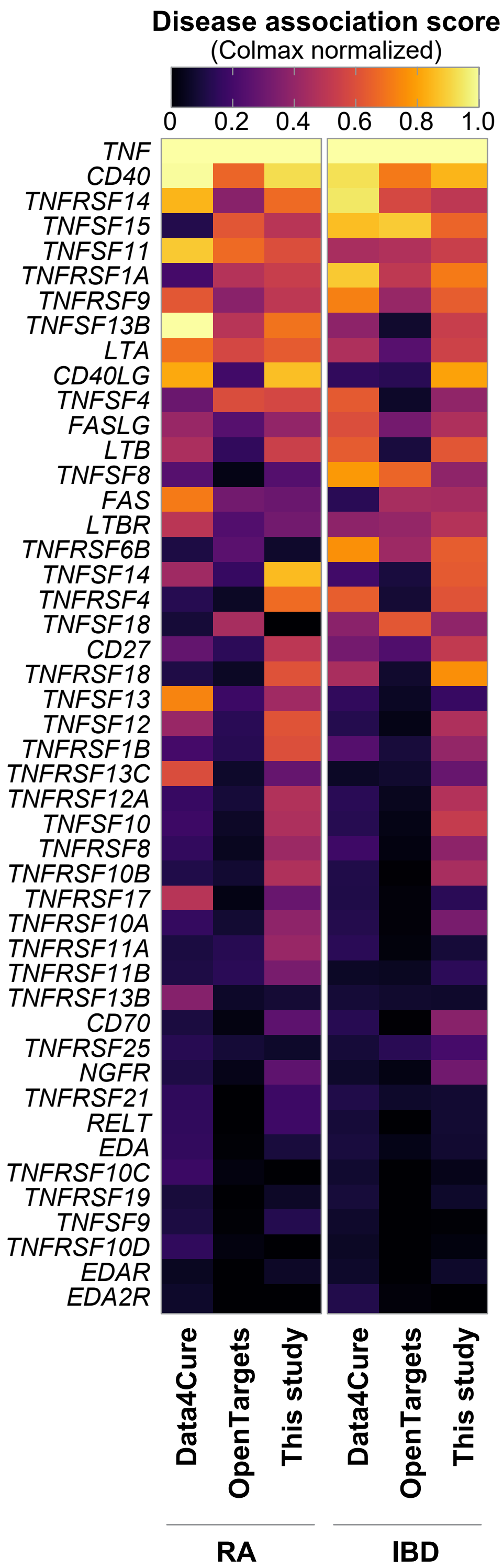
Disease association score
(Colmax normalized)
0
0.2
0.4
0.6
0.8
1.0
TNF
CD40
TNFRSF14
TNFSF15
TNFSF11
TNFRSF1A
TNFRSF9
TNFSF13B
LTA
CD40LG
TNFSF4
FASLG
LTB
TNFSF8
FAS
LTBR
TNFRSF6B
TNFSF14
TNFRSF4
TNFSF18
CD27
TNFRSF18
TNFSF13
TNFSF12
TNFRSF1B
TNFRSF13C
TNFRSF12A
TNFSF10
TNFRSF8
TNFRSF10B
TNFRSF17
TNFRSF10A
TNFRSF11A
TNFRSF11B
TNFRSF13B
CD70
TNFRSF25
NGFR
TNFRSF21
RELT
EDA
TNFRSF10C
TNFRSF19
TNFSF9
TNFRSF10D
EDAR
EDA2R
Data4Cure
OpenTargets
This study
Data4Cure
OpenTargets
This study
RA
IBD

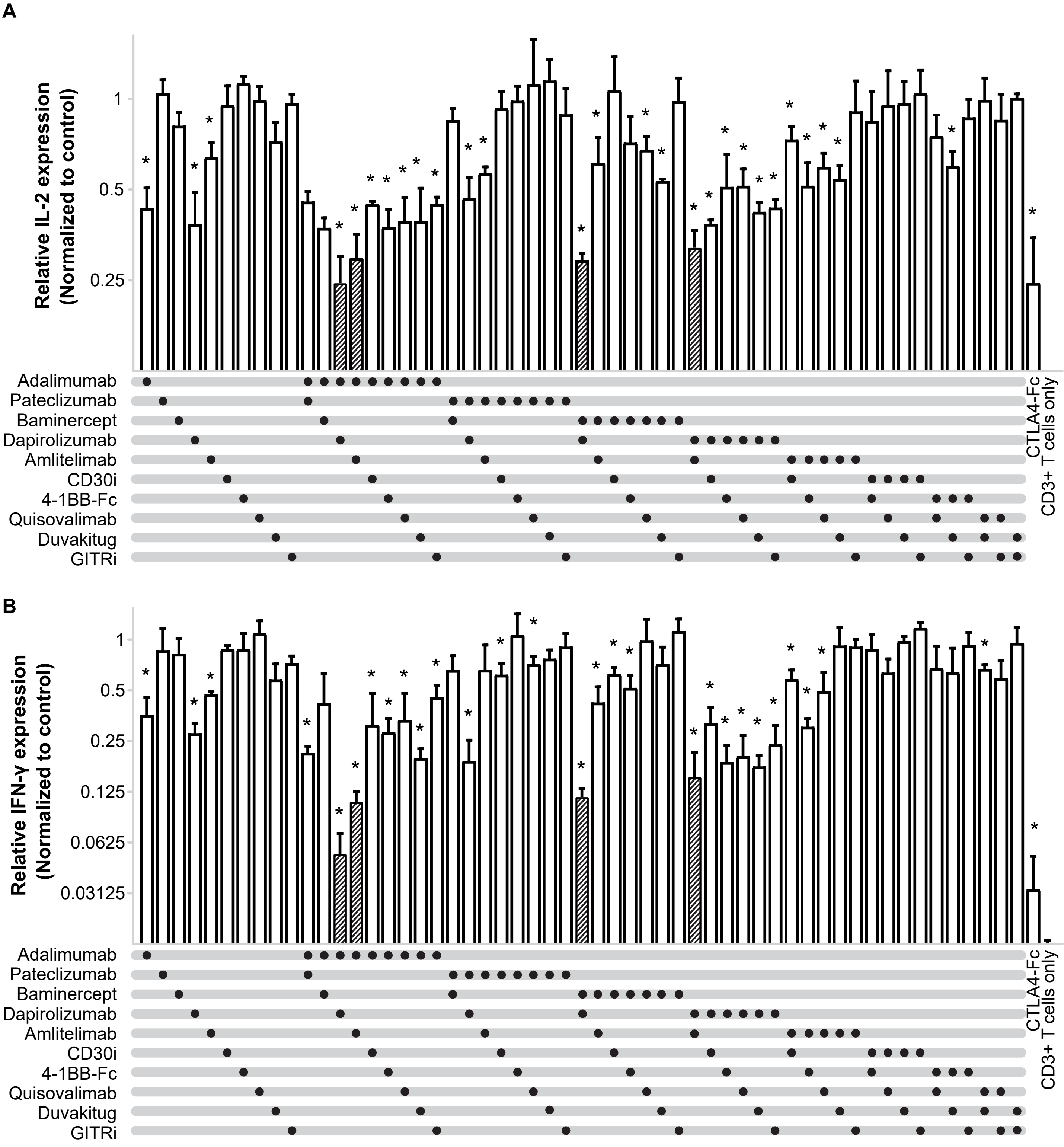
A
Relative IL-2 expression (Normalized to control)
1
0.5
0.25
Adalimumab
Pateclizumab
Baminercept
Dapirolizumab
Amlitelimab
CD30i
4-1BB-Fc
Quisovalimab
Duvakitug
GITRi
CTLA4-Fc
CD3+ T cells only
B
Relative IFN-γ expression (Normalized to control)
1
0.5
0.25
0.125
0.0625
0.03125

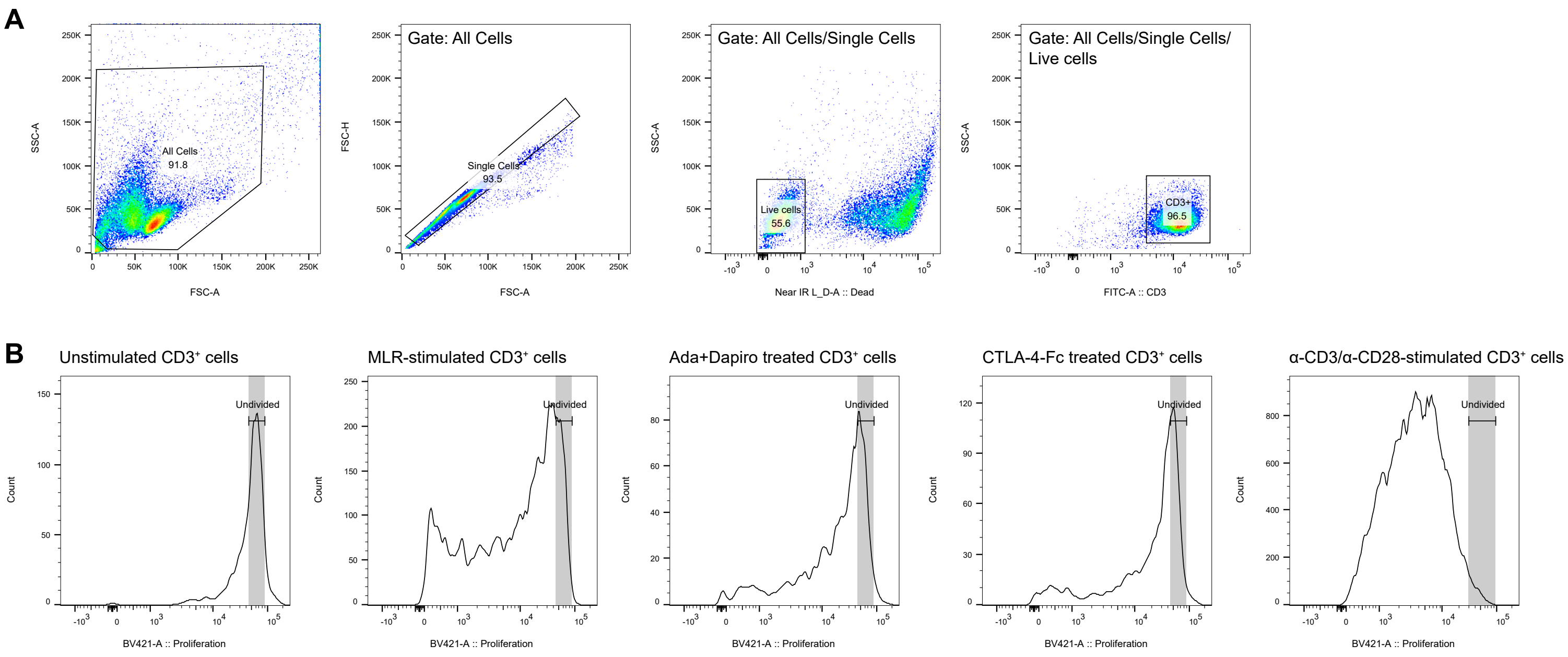
A
All Cells
91.8
SSC-A
FSC-A
Gate: All Cells
Single Cells
93.5
FSC-H
FSC-A
Gate: All Cells/Single Cells
Live cells
55.6
SSC-A
Near IR L_D-A :: Dead
Gate: All Cells/Single Cells/
Live cells
CD3+
96.5
SSC-A
FITC-A :: CD3
B
Unstimulated CD3⁺ cells
MLR-stimulated CD3⁺ cells
Ada+Dapiro treated CD3⁺ cells
CTLA-4-Fc treated CD3⁺ cells
α-CD3/α-CD28-stimulated CD3⁺ cells
Undivided
Count
BV421-A :: Proliferation

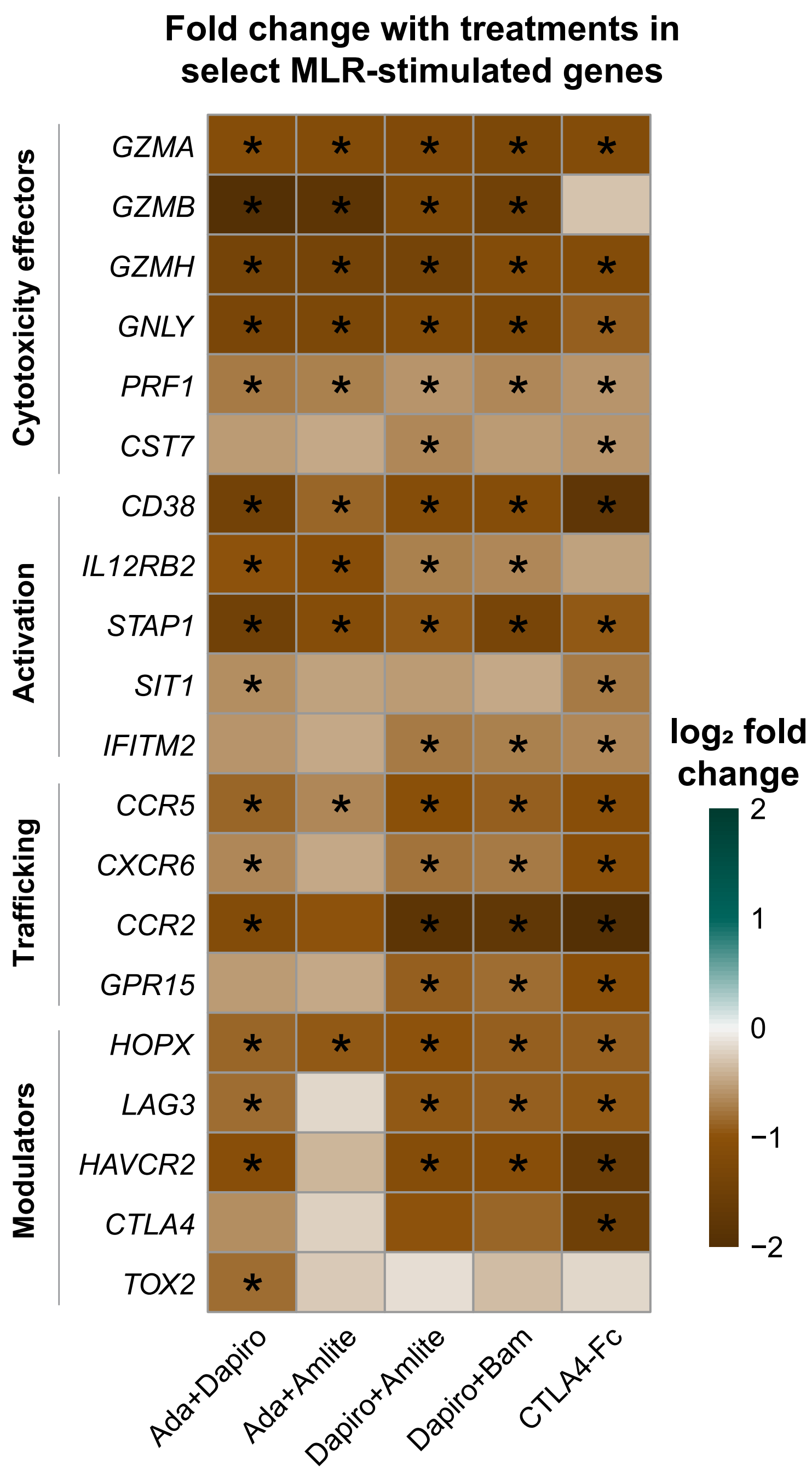

Fold change with treatments in select MLR-stimulated genes
Cytotoxicity effectors
Activation
Trafficking
Modulators
GZMA
GZMB
GZMH
GNLY
PRF1
CST7
CD38
IL12RB2
STAP1
SIT1
IFITM2
CCR5
CXCR6
CCR2
GPR15
HOPX
LAG3
HAVCR2
CTLA4
TOX2
Ada+Dapiro
Ada+Amlite
Dapiro+Amlite
Dapiro+Bam
CTLA4-Fc
log₂ fold change
2
1
0
−1
−2

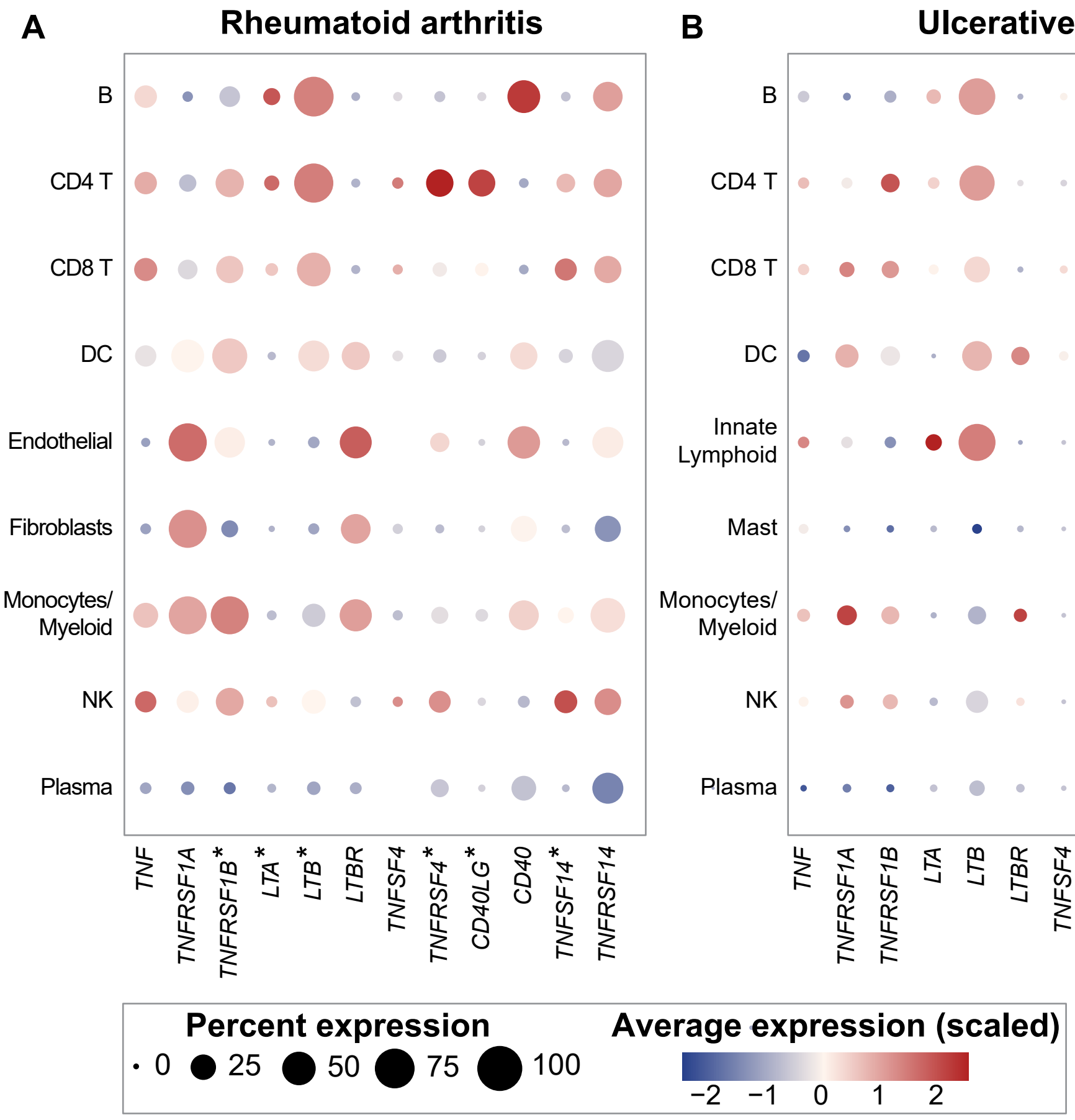
A
Rheumatoid arthritis
B
Ulcerative colitis
Crohn's disease
B
CD4 T
CD8 T
DC
Endothelial
Fibroblasts
Monocytes/ Myeloid
NK
Plasma
Innate Lymphoid
Mast
TNF
TNFRSF1A
TNFRSF1B*
LTA*
LTB*
LTBR
TNFSF4
TNFRSF4*
CD40LG*
CD40
TNFSF14*
TNFRSF14
TNFRSF1B#
LTBR#
TNFSF4#
Percent expression
0
25
50
75
100
Average expression (scaled)
−2
−1
0
1
2
